# Application of Granular Biocomposites Based on Homogenised Peat for Absorption of Oil Products

**DOI:** 10.3390/ma15041306

**Published:** 2022-02-10

**Authors:** Kristine Irtiseva, Marika Mosina, Anastasija Tumilovica, Vjaceslavs Lapkovskis, Viktors Mironovs, Jurijs Ozolins, Valentina Stepanova, Andrei Shishkin

**Affiliations:** 1 Rudolfs Cimdins Riga Biomaterials Innovations and Development Centre of RTU, Institute of General Chemical Engineering, Faculty of Materials Science and Applied Chemistry, Riga Technical University, Pulka 3, LV-1007 Riga, Latvia; kristine.irtiseva@rtu.lv (K.I.); marika.mosina@rtu.lv (M.M.); anastasija.tumilovica@rtu.lv (A.T.); jurijs.ozolins@rtu.lv (J.O.); valentina.stepanova@rtu.lv (V.S.); 2 Scientific Laboratory of Powder Materials, Faculty of Mechanical Engineering, Riga Technical University, 6B Kipsalas Street, Lab 319, LV-1048 Riga, Latvia; vjaceslavs.lapkovskis@rtu.lv (V.L.); viktors.mironovs@rtu.lv (V.M.); 3 Institute of Aeronautics, Riga Technical University, 6B Kipsalas Street, LV-1048 Riga, Latvia

**Keywords:** sorption, kinetics, peat, cenosphere, oil sorption, Weber–Morris, diffusion model, granules, biocomposite

## Abstract

Among the various methods for collecting oil spills and oil products, including from the water surface, one of the most effective is the use of sorbents. In this work, three-component bio-based composite granular adsorbents were produced and studied for oil products’ pollution collection. A bio-based binder made of peat, devulcanised crumb rubber from used tyres, and part fly ash as cenospheres were used for absorbent production. The structure, surface morphology, porosity, mechanical properties, and sorption kinetics of the obtained samples were studied. Composite hydrophobicity and sorption capacity to oil products, such as diesel fuel (DF) and motor oil (MO), were determined. The obtained pellets are characterised by a sufficiently pronounced ability to absorb oil products such as DF. As the amount of CR in the granules increases, the diesel absorption capacity increases significantly. The case of 30-70-0 is almost three times higher than the granules from homogenised peat. The increase in q is due to two factors: the pronounced surface hydrophobicity of the samples (Θ = 152°) and a heterogeneous porous granule structure. The presence of the cenosphere in the biocomposite reduces its surface hydrophobicity while increasing the diesel absorption capacity. Relatively rapid realisation of the maximum saturation by the MO was noted. In common, the designed absorbent shows up to 0.7 g·g^−1^ sorption capacity for MO and up to 1.55 g·g^−1^ sorption capacity for diesel. A possible mechanism of DF absorption and the limiting stages of the process approximated for different kinetic models are discussed. The Weber–Morris diffusion model is used to primarily distinguish the limiting effect of the external and internal diffusion of the adsorbate on the absorption process.

## 1. Introduction

One of the basic principles of the circular economy is the maximum economy of raw materials and the maximum use as well as processing of secondary raw materials. A large amount of waste is the cenospheres (CS), which are generated from the combustion of coal in coal-fired power plants [1], and waste rubber in the form of crumb rubber (CR) [2,3] from used tyres. By using peat processing products as absorbers from CS and crushed rubber, it is possible to obtain sufficiently porous biocomposites with specific properties, including the ability to absorb oil products on their surface [4,5]. Among the various methods for collecting oil spills and oil products, including from the water surface, one of the most effective is the use of sorbents. Several requirements are imposed on the sorbents used to collect oil products from the water surface: the sorbent should mainly absorb oil products upon contact with the water surface, the maximum saturation of the sorbent with oil should be reached as soon as possible after contact, and afterwards the sorbent should remain on the surface for a long time. Thus, the effectiveness of sorbents for the absorption of oil products from the water surface is assessed by three criteria: the absorption capacity of oil products, water absorption capacity, and buoyancy of the sorbent [4,6].

Commercial adsorbents are effective but expensive. Natural sorbents quickly absorb water and do not selectively absorb the oil product or present a good-enough buoyancy. To minimise water absorption and hydrophilicity, adsorbents need to be modified with hydrophobic agents. An increasing number of oil spill incidents call for an increased necessity for cheaper absorbents made of secondary materials.

Liu et al. [7] reported the hydrophobic plant fibre sponge preparation via a simple treatment by hexamethyldisilazane vapours. The material is characterised by high porosity, high mechanical durability, excellent hydrophobicity with a water contact angle of 145°, high oil absorptive capacity (15–40 g·g^−1^), and good selectivity [7]. Wu and Zhou [8] reported waste tyre rubber (WTR) prepared by the graft copolymerisation-blending method using WTR and 4-tert-butyl styrene as monomers, and divinylbenzene and benzoyl peroxide were employed as a crosslinker and initiator, respectively. Oil absorptive material has a maximum absorption capacity up to 24.0 g·g^−1^. Oil removal from the aqueous state by natural fibrous sorbents has been widely studied [9]. The peat and peat treatment products have also been widely studied for absorbent design [10]. Alameri et al. [11] investigated the use of peat-derived biochar as a bio-sorbent for the sorption and removal of crude oil spills from synthetic seawater, with up to 32.5 g·g^−1^ sorption capacity per crude oil.

For practical application, it is necessary to focus on low-cost (preferable secondary) raw material use, maximal sorption capacity and buoyancy, the ability to collect the absorbent from the water surface, and oil sorption kinetics to collect the spill in a short time.

Table 1 summarises several examples of developed and studied sorbents for crude oil and oil products’ spill clean-up and water treatments. As can be seen, sorbents with high sorption capacity require deep chemical and thermal treatments. As a result, they are not eco-friendly, using hazardous and toxic chemicals [7,8,12,13]; are not sustainable, as they produce a lot of wastewater water [8,13] and gases [14]; and have a high cost [8,12] due to the complex production and used equipment.

The investigated and described sorbents without deep chemical treatment have significantly lower sorption values. However, the significant disadvantage of the described sorbents (by except [8]) is the hardly suitable practical application in real spill collection: spreading and collecting such sorbents from the water surface due to their form/shape. Additionally, according to the literature, the maximal sorption capacity of the oil products is mainly noted starting from 5 and to a longer time: time can be 5–10 min [16], 20–30 min [17], and 30 min [8].

A previous work [4] performed manufacturing and characterisation of a three-component bio-based composite material. The key components used were a bio-based binder made of peat, devulcanised CR from used tyres, and part of the fly ash a cenospheres. The material was prepared in a block to investigate its mechanical properties and density. Preliminary studies to determine the sorption of water and oil products (in the form of granules) demonstrated promising results. Individual investigation for the granulated material for oil products is necessary because it describes its properties as products–granules but not as a powder-like or sponge material that most of the papers usually describe.

In this work, granular adsorbents from secondary raw materials were produced and studied for oil products’ pollution collection. The structure, surface morphology, inner structure of the granules (by mCT), porosity, mechanical properties, and sorption kinetics of the obtained samples were analysed. Their hydrophobicity and sorption capacity were determined in relation to oil products: diesel fuel (DF) and motor oil (MO).

## 2. Materials and Methods

### 2.1. Materials and Composites Used

**Figure 1:**
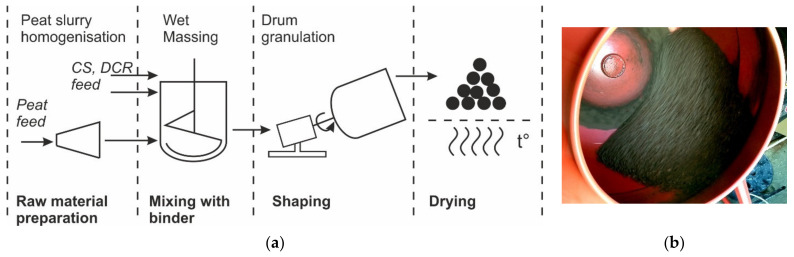
Production scheme (**a**) and used drum granulator (**b**) in operation.

Using the wet granulation method, nineteen granule compositions were prepared. The composition of the granular composites before drying is shown in Table 2 and Table 3. They are designated CR-HP-CS, where CR is a percentage by the mass of devulcanised rubber, HP is a percentage by the mass of homogenised peat (including water), and CS is a percentage by the mass of cenospheres. The composition changes significantly after drying due to the evaporation of water from the absorbent. The composition after drying is also presented in Table 2 and Table 3 but for convenience, the initial compositions’ (including water from HP) designations are used. The detailed granules production method has been published in a previous work [4]. The production scheme for granular adsorbents and the used drum granulator are represented in Figure 1a,b respectively.

### 2.2. Investigations of the Surface Morphology and Structure of Samples

The morphology of the granule surface was studied using a Kyence VHX-1000 digital microscope (Keyence Corp. Osaka, Japan). The Kyence VHX-1000 digital microscope operates in a magnification range from 0 to 200 times and was equipped with VH-Z20R/W lenses. The microscope allows you to study the under-study object’s topography from different angles, which will enable you to obtain a three-dimensional picture.

The Tescan Mira/LMU scanning electron microscope (Tescan, Brno, Czech Republic) was used to study the microstructure of the sample surface and visually assess the pore morphology. Before analysis, the samples were coated with a 10–15 nm layer of gold using an Emitech K550X (Emitech, Montigny-le-Bretonneux, France).

The internal structure of the samples was assessed using X-ray micro-computed tomography (microCT). The cycle continued up to 180 degrees. The resulting images were combined to obtain a three-dimensional image during the reconstruction process. MicroCt 50, Scanco Medical (Scanco Medical AG, Wangen-Brüttisellen, Switzerland) was used. Colourless MicroCT scans were made using cabinet cone-beam microCT (µCT50, Scanco Medical AG, Wangen-Bruttisellen, Switzerland) with the settings: 70 kVp energy; 114 μA tube current; 0.5 mm Al filter; 1005 ms integration time; and 5 µm voxel size. Each sample was scanned for 11 h. Reconstruction of 3D datasets from the microCT projection data, including beam-hardening correction, was performed automatically after completing each cone-beam image stack using the SCANCO GPU (Scanco Medical AG, Wangen-Bruttisellen, Switzerland) Accelerated Reconstruction System. The visualisation module performs sophisticated 3D rendering of large datasets using high-quality ray-tracing algorithms.

### 2.3. Mechanical Compressive Strength

The strength of the composite granules was determined using a KAHL mechanical strength tester (Amandus Kahl GmbH & Co. KG, Reinbek, Deutschland). The principle of operation of the device is to fix the force required to compress the granule. The compression of the granules took place at a compression speed of 0.5 mm·min^−1^. For the study of each composition, 30–40 granules with similar geometry and dimensions of 6.0 ± 0.5 mm were tested.

### 2.4. Nitrogen Adsorption Porosimetry

Nitrogen adsorption porosimetry can be used to determine the specific surface area of a sample, total pore volume, and pore size distribution and scatter. The Brunauer–Emmett–Teller (BET) surface analysis method is based on the adsorption and desorption of nitrogen. Samples were degassed in an Autosorb Degasser Model AD-9 (Anton Paar, St Albans, England) before nitrogen adsorption. Degassing is performed to remove excess moisture and impurities from the samples. The samples were degassed for 24 h at 100 °C. The samples were weighed before and after degassing. Higher temperatures speed up the degassing process but can only be used if they do not affect the structure of the sample.

Nitrogen sorption was performed on a QUADRASORB SI (Quantachrome Corporation, Boynton Beach, FL, US) device. The measurement was carried out at a temperature when nitrogen is in a liquid state (−196.15 °C). During this process, nitrogen molecules are physically adsorbed onto the sample surface. When determining the amount of adsorbed gas as a function of pressure under isothermal conditions, adsorption isotherms are constructed, after which the distribution of pores in the material is determined.

### 2.5. Surface Energy and Contact Angle

If a drop of liquid gets on a hard surface, its ability to wet the surface can be characterised by the wetting angle or contact angle. As the surface hydrophobicity increases, the contact angle values increase. In the case of a highly hydrophobic surface, the contact angle is 150° or more. The contact surface and the liquid contact angle of the obtained biocomposites made in blocks were determined using the drop method with the optical tensiometer Theta (Attention, Helsinki, Finland,). Distilled water was used in the experiment, the volume of the liquid drop was 5 μL, and 5 parallel measurements were performed for each sample.

### 2.6. Absorption Capacity of Oil Products

The absorption capacity of oil products was determined for granules of biocomposites by combining various compositions of peat, CS, and CR. For sorption studies, the liquid media-oil products, namely diesel fuel miles PLUS (EN950 standard, by Circle-K Latvia Sia) with a density of 0.815 g·cm^−3^ and motor oil LazerWay 5-W40 (by Circle-K Latvia Sia) with a density of 0.835 g·cm^−3^, were used. The ability of the pellets to absorb DF and MO [21,22] was determined using the gravimetric method and samples weighing 1.0 ± 0.1 g were placed in a stainless-steel mesh and incubated once in one of these media. Measurements were taken after 1, 3, 5, and 10 min. The absorption capacity of oil products was performed by immersion of a single granule (diameter of 5–8 mm) in the metal mesh into a 2 mm thick layer of the studied oil product in a Petri dish, which imitated an oil spill. Each granule was extracted from the Petri dish by the exact time gap for weighting. The exceeding layer or droplets (if any) was wiped by a wet (wetted by the same oil product) paper towel from the metal mesh and from the granules. Since the granules extraction moment sorption time counting is paused until it returns to the Petri dish. The measurements were then repeated every 5 min until equilibrium was reached and the weight of the saturated granules remained unchanged. The mass of the granules was recorded with an accuracy of 0.001 g. For each composition, 6 parallel measurements were performed. The astringency of the oil products was determined by Equation (1) [23,24]:

q = (m_t_ − m_0_ − m_s_)/m_0_ (1)

where

q—absorption capacity (g·g^−1^);

m_t_—the mass of the sample and mesh after a certain period of time (g);

m_s_—mesh mass (g); and

m_0_—initial mass of the sample (g).

## 3. Results

Biocomposites based on HP slurry processing products were obtained using CS and CR. For the obtained biocomposites in the form of granules, the influence of the composition of CR and CS on their mechanical properties, surface morphology, structure, and hydrophobicity was determined. The ability of the obtained granular biocomposites to absorb oil products to their surface was experimentally determined using DF and MO.

**Figure 2:**
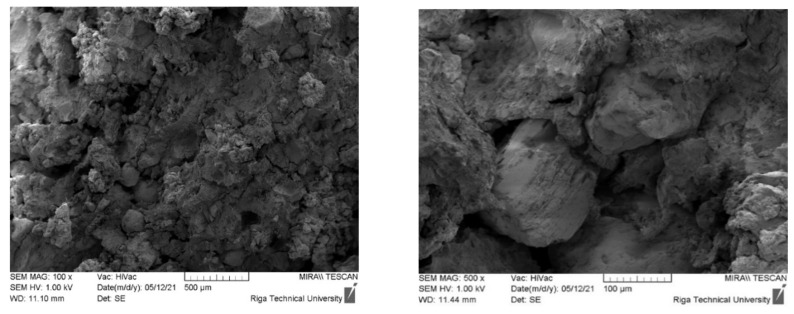
Sample images of samples 5-95-0 after loading taken at ×100 and ×500 magnification. The yellow arrows indicate CR particles.

As shown in a previous publication [4], as the content of CR in the biocomposite increases, its compressive strength decreases significantly compared to the compressive strength of a block of dried homogenised peat. An increase in the mass fraction of CR in the original composition from 5% to 30% reduces the mechanical compressive strength from 8 MPa to 2 MPa. In this case, the apparent density of the samples decreased from 1.20 g·cm^−3^ (5 wt.% CR) to 0.95 g·cm^−3^ (30 wt.% CR). Information on the nature of the destruction of samples can be obtained by analysing their SEM images after the loading of the sample. As shown from Figure 2, CR is evenly distributed in the sample volume, which was also demonstrated by digital microscopic images. Cracking and failure of the sample under load occurred at the interface between the aggregate and the absorbent, which can be explained by the insufficient interconnection or adhesion of the CR and HP matrix.

When oil and oil products are collected from the water surface using porous sorbents, the process of their absorption is usually physical. The number of contaminants depends on the hydrophobicity, oliophility, and specific surface area of the material. The contact angle was determined for the test samples by the drop method.

**Figure 3:**
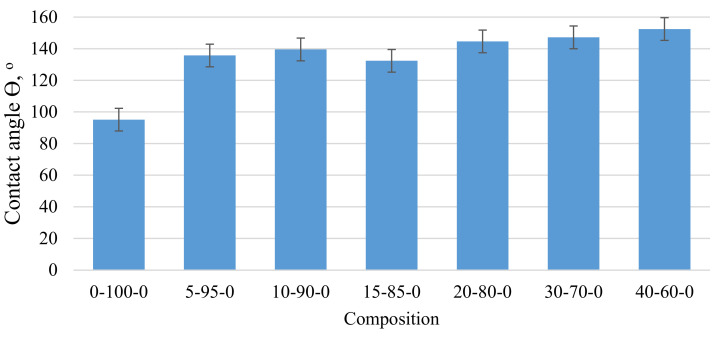
Variation of the wetting angle of the biocomposite depending on the amount of CR in the composite.

As can be seen from Figure 3, with an increase in the mass fraction of CR in the composite material, the contact angle with water also significantly increases, which indicates that the addition of CR increases the hydrophobicity of the samples. Thus, replacing 5 wt.% peat with CR can show a 42% increase in contact angle. As the amount of CR in the composite increases, the contact angle tends to increase. The most significant contact angle (Θ) 152° was observed for sample 40-60-0. In this case, the sample was barely wetted by water and the drops tended to drain off the surface. The hydrophobicity of the surface of the samples should facilitate better absorption of oil products.

To increase the buoyancy of the composition, the weight of CS was introduced. Additionally, in this case, the addition of CS, as in the case of CR, reduces the compressive strength of the composite compared to the strength of the HP block and strength of the composite blocks containing CR, as shown in Table 4.

The decrease in the mechanical strength of the biocomposite can be explained by the formation of additional voids and structural defects when introduced into the CS system, as evidenced by the decrease in the apparent density ρ (g·cm^−3^) of the samples to 0.65 g·cm^−3^ (5-85-10).

**Figure 4:**
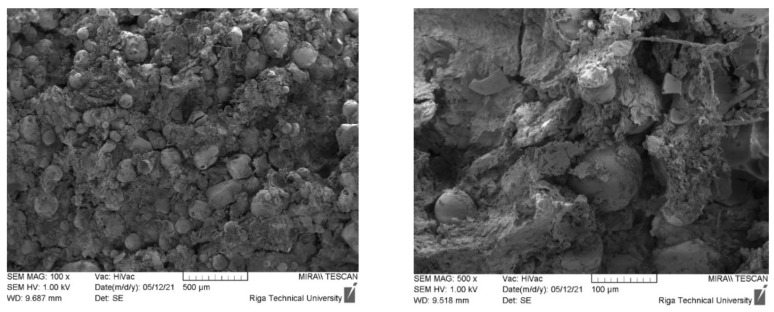
Sample images of samples 5-90-5 after destruction taken under ×100 and ×500 magnification.

By analysing the SEM images of the samples (Figure 4), it can be concluded that, similarly to the compositions containing CR, in the compositions containing CR and CS, their destruction by compression occurs at the interface between the homogenised peat and the aggregates. In addition, the introduction of CS into the system increases the proportion of structural defects and voids.

The incorporation of CS into the composite was expected to reduce the surface hydrophobicity of the samples. As shown from the Table 4 data, this tendency was observed. Still, the experimentally determined values of the wetting angle Θ are sufficiently high, that is, not less than 120°, which is typical for hydrophobic surfaces.

**Figure 5:**
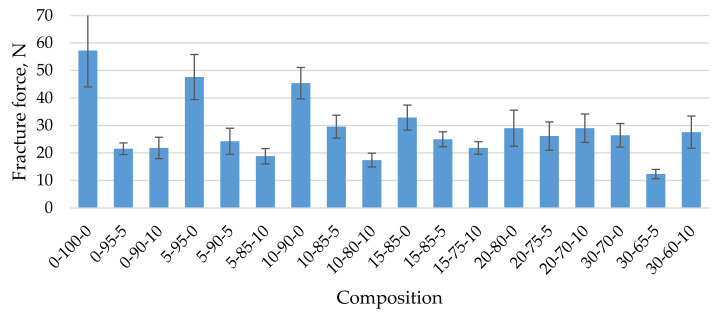
Mechanical strength of biocomposite granules depending on their composition.

**Figure 6:**
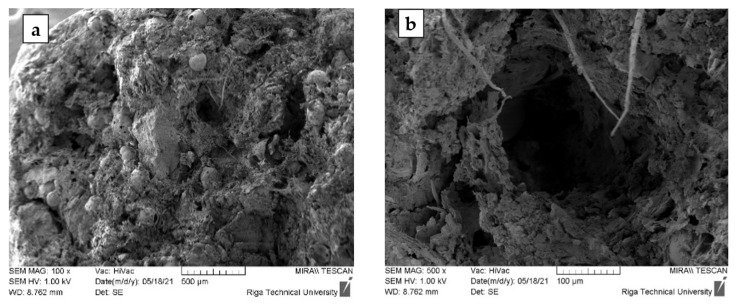
Granules with a composition of 5-90-5 SEM images at ×100 (**a**) and ×500 (**b**) magnification.

**Figure 7:**
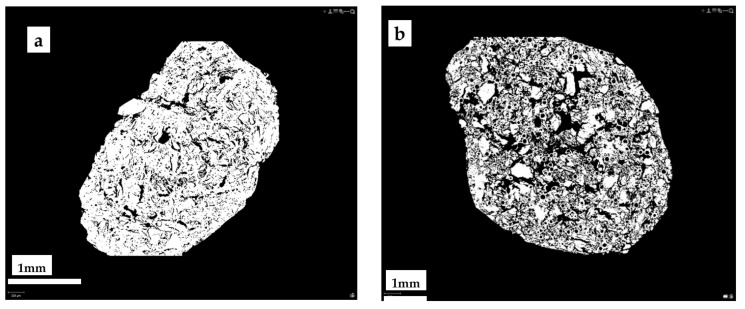
Photomicrographs of MCt cross-sections of granules 0-100-0 (**a**) and 5-90-5 (**b**).

To use the studied biocomposites as sorbents for the absorption of oil products, they were obtained in the form of granules using the granulation technology described in the publication. An essential property of granules for their successful use is mechanical strength. Figure 5 shows the mechanical strength of biocomposite granules when compressed into different granule compositions. As the concentration of CR in the composite increases, the mechanical strength of the granules decreases from 48 N on average if the composition contains 5% CR to 26 N if the composition contains 30% CR (Figure 5). As already noted, a similar trend was observed for biocomposites in the form of a block. In this case, the mechanical strength of the composite decreased, on average, four times with similar increasing CR concentrations. The incorporation of cenospheres into the composition further reduced the mechanical compressive strength of the granules, which may be due to insufficient bonding of the microspheres and homogenised peat, on the one hand, and a high degree of composite filling in the case of dried granules (average 44.2 wt.% 5-90-5 and up to 79.3% by weight in the case of 30-65-5), which of course affects the mechanical strength of the product. The observed changes in the mechanical strength of the granules are also related to the occurrence of structural defects, pores, and voids in the granulation process (Figure 5, Figure 6 and Figure 7).

The addition of CS to the biocomposite composition promotes and improves the buoyancy of the granules, which is very important when using it for collecting oil products from the water surface. Granules containing 5 to 30 wt.% of the CR are not buoyant. In turn, granules obtained from the initial mixture, which, without CR, additionally contains from 5 to 10 wt.% CS, show good buoyancy. Granules from the initial mixture with different CR content and 10 wt.% CS remain buoyant for more than 10 days, making it possible to collect them from the water surface after the absorption of oil products.

One of the most critical parameters characterising sorbents is their porosity and specific surface area. As shown in Table 5, the porosity of the biocomposite granules was significantly increased when CR and CS were added to the starting mixture.

In addition, the data obtained by the BET method are close to the data obtained by the MCt analysis. The porosity of granules with a composition of 5-90-5 increases practically two times compared to the granules obtained from the homogenised peat. At the same time, of course, the apparent density of the samples decreases and the specific surface area of the material increases to 0.702 m^2^·g^−1^.

The SEM microphotographs of samples 5-90-5 (Figure 6) show a distinctly porous structure with characteristic cylindrical voids formed during the granulation process in the rotary granulator. X-ray microtomography (MCt) data provide more objective information on the internal structure of the granular material. Figure 7 shows the cross-sections of the samples. A sufficiently homogeneous structure with small voids due to granulation was observed in the cross-section of sample 0-100-0 (a). In the case of sample 5-90-5 (b), on the other hand, a markedly heterogeneous structure was seen in which CR and CS particles, as well as voids between the particles and the homogenised peat, appear uniform in volume. The heterogeneous structure resulting from the granulation process determines the porosity and specific surface area of the obtained material, which is practically twice as large as in the sample 0-100-0 (Table 5). Increasing the amount of CR and CS in the samples increases the structural heterogeneity and porosity.

**Figure 8:**
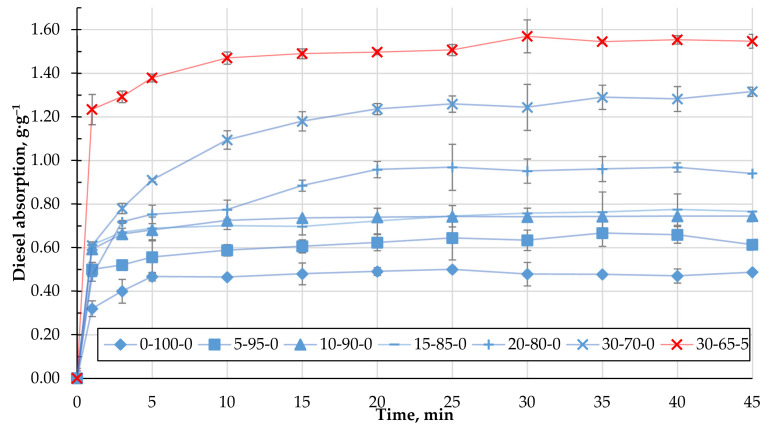
Adsorption kinetics of DF with biocomposite pellets containing CR.

**Figure 9:**
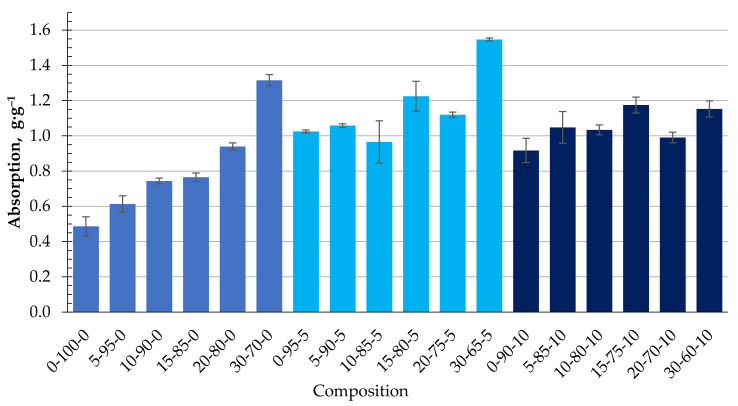
Absorption capacity (g·g^−1^) of DF depending on the composition of the composite.

The ability of the obtained biocomposite to absorb oil products, namely DF and MO, was studied. Figure 8 shows the experimentally obtained kinetics of DF absorption using the studied biocomposite granules containing CR. As can be seen, with an increasing amount of CR in the composition of the granules, the DF absorption capacity increases significantly and, in the case of 30-70-0, is almost three times higher than the granules from the homogenised peat. The increase in q is due to two factors: pronounced surface hydrophobicity of the samples (Θ = 152°) and non-uniform porous granule structure. Even though the presence of the cenosphere content in the biocomposite reduces its surface hydrophobicity (Table 4), the DF absorption capacity increases, as shown in Figure 9. This condition could be related to the sufficiently developed porous granule’s structure that occurs during their formation in the rotary granulator. Among the studied compositions, granules with a composition of 30-65-5 stand out, in which case the average DF absorption capacity reaches 1.5 g·g^−1^.

**Figure 10:**
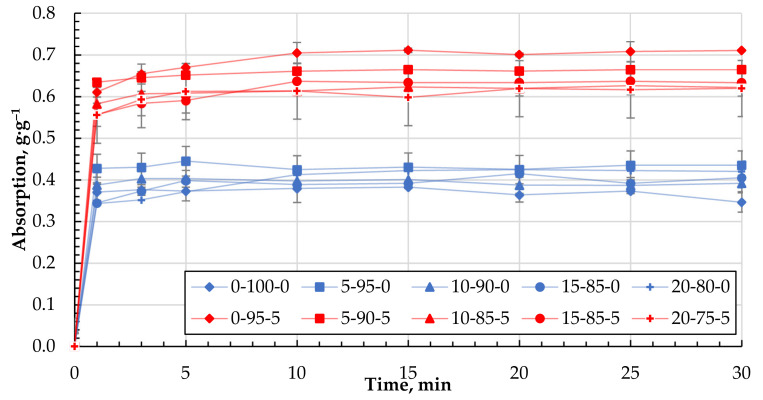
Absorption kinetics of MO for granules with 0 and 5 wt.% of CS.

**Figure 11:**
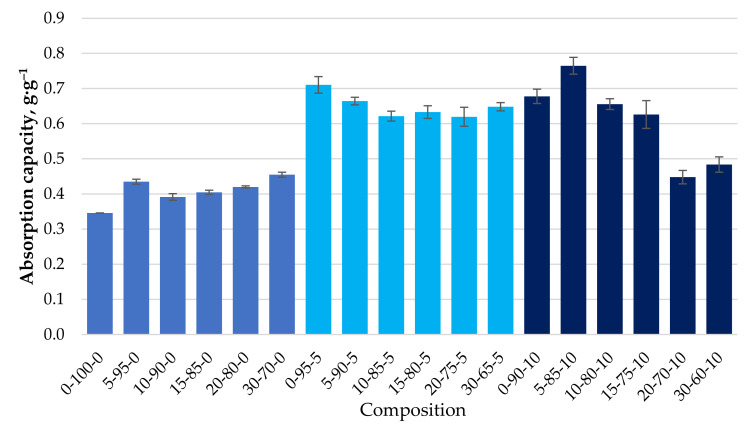
Absorption capacity (g·g^−1^) of MO depending on the composition of the composite.

Their ability to bind motor oil, with a viscosity much higher than DF, was also determined for the different granule compositions obtained. For all formulations, the maximum rate of MO retention was achieved in the first minutes of contact and practically did not increase with increasing residence time (Figure 10). This indicates a different mechanism of retention of MO by the biocomposite granules compared to that of diesel fuel. Figure 11 shows the maximum degree of MO retention achieved for all the granule formulations tested. The introduction of CR particles into the composition contributes to motor oil binding insignificantly. At the same time, the use of CS, additionally, as a filler increases the ability to bind motor oil on average by 38% compared with granules containing only CR and reaches the value of 0.65 ± 0.05 g·g^−1^.

## 4. Discussion

To be able to determine the possible mechanism of DF absorption and the limiting stages of the process, the experimental data can be satisfactorily approximated for different kinetic models. To primarily distinguish the limiting effect of external and internal diffusion of the adsorbate on the absorption process, the Weber–Morris diffusion model [25] can be used, the mathematical expression of which is Equation (2):

q_t_ = K_id_t^0.5^ + C (2)

where

q_t_—amount of adsorbed substance in g·g^−1^;

K_id_—Weber–Morris constant or internal diffusion rate constant in pores in g·g^−1·^min^0.5^; and

C—a constant parameter related to the boundary layer thickness in g·g^−1^.

**Figure 12:**
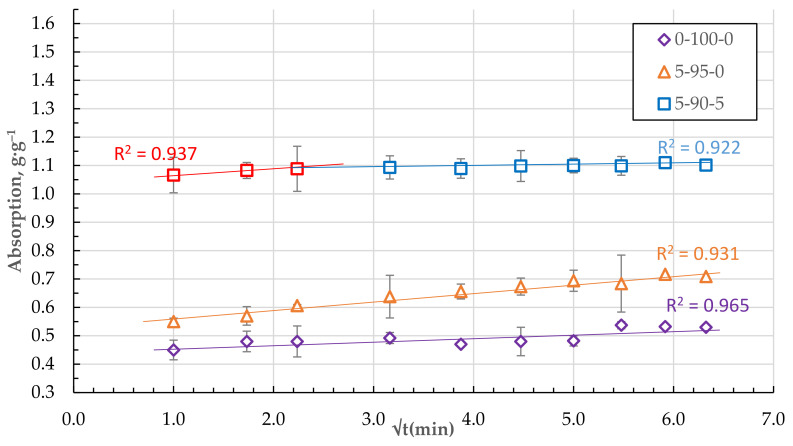
Absorption of DF in q_t_ − t^0.5^ coordinates for the granules with 5 and 0 wt.% of CSand HP only.

As shown in Figure 12, the absorption relationships of granules from pure, homogenised peat and DF with a small amount of CR (composition 0-100-0 and 5-95-0) in q_t_ − t^0.5^ coordinates are practically liberal. It is believed that the diffusion of a substance into the sorbent pores or internal diffusion is a limiting step in the rate of the adsorption process if the relation qt = f (t^0.5^) is linear and crosses the origin of the coordinate (C = 0). In cases where the constant C is not zero, the rate of adsorption is significantly influenced by the absorption of the sorbate to the surface of the granules and the diffusion through the boundary layer [25,26].

**Figure 13:**
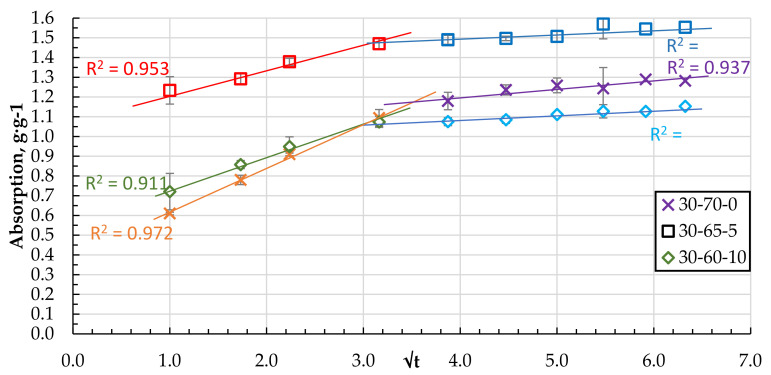
Absorption of DF in q_t_ − t^0.5^ coordinates for the granules with 0, 5, and 10 wt.% of CS.

As the number of components increases, the granular structure of the biocomposite becomes more porous with pronounced defects and voids. Two stages of sorbate mass transfer can be distinguished in the qt − t^0.5^ coordinates of the DF absorption process (see Figure 12 and Figure 13).

The steepest stage of the relationship q_t_ − t^0.5^ characterises the mass transfer from the volume of DF medium to the sorbent surface or external diffusion. The other less steep (see Figure 12 and Figure 13) stage can be attributed to the transport and absorption of DF molecules in the pores of the sorbent or the so-called internal diffusion [25,27,28,29,30]. The point of transition to the relation q_t_ = f (t^0.5^), which could correspond to the saturation of the sorbent granule surface, is reached for all compositions, on average, at the same time from 5 to 10 min.

In the case of MO, as demonstrated above, the saturation of the sorbent is reached in the first minutes (Figure 10) and, most likely, in this case, MO binding mainly occurs only by the surface of the granule. Due to the increased viscosity of MO, its diffusive penetration into the pores of the sorbent practically does not occur. The state of the sorbent surface would determine the ability to bind MO. Sorbents containing particles of CR and cenospheres are characterised by a more developed specific surface and porosity (Table 4 and Figure 6 and Figure 7), which contributes to the increase of MO binding by the granule surface. It is particularly evident for composites containing CS. With good filler content, in our case, the actual mass content of CS in the dried sample 0-95-5 is 27.3% and in sample 0-90-10 is 44.2% (Table 2), while numerous channels, voids, and structural defects can be formed in the near-surface layers and filled with MO. This may lead to a significant increase in the oil absorption of the sorbents (Figure 11).

**Figure 14:**
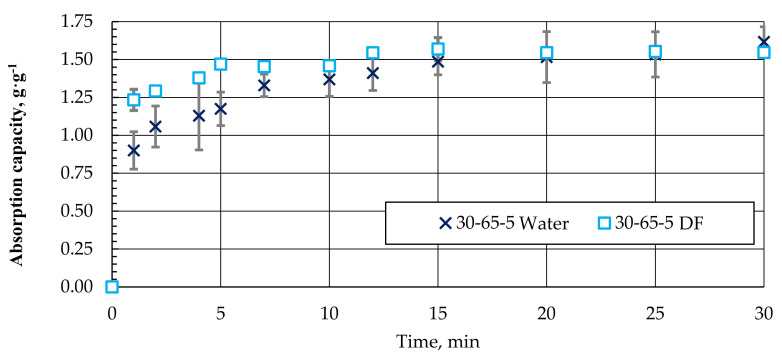
Absorption kinetics (approximation) of water and DF for granules with a composition of 30-65-5.

When using granules to collect oil products from the water surface, they must absorb water to a minimum. As the surface hydrophobicity increased, the ability of the samples to absorb water was expected to decrease. However, as the experiment has shown, pellets with different compositions absorb practically as much water as the g·g^−1^ of DF. A comparative example of water and DF absorption kinetics for granules with a composition of 30-65-5 is shown in Figure 14.

As can be seen, the nature of the absorption of water and DF in the biocomposite pellets is similar. It is only necessary to note that DF absorption and saturation are relatively faster. Obviously, due to the hydrophobicity of the surface, the DF absorption process could be a priority in the case of a water–DF mixture.

When studying the process of MO absorption using the obtained sorbents, it was found that the maximum degree of saturation is already reached in the first minutes of the absorption process (Figure 10). By increasing the time of the absorption process, the achieved degree of saturation for the studied biocomposites barely changes. This indicates that the oil absorption process mainly takes place on the surface of the sorbent. Due to the increased viscosity of the oil, internal diffusion of the products is minimal or does not occur.

Depending on the composition of the biocomposite, there are no apparent differences in the maximum degree of the sorption of MO, which ranges from 0.4 ± 0.05 g·g^−1^ for compositions without CS to 0.65 ± 0.05 g·g^−1^ for compositions with CS 27-11 wt.% in a dry mixture (please see Table 2 and Table 3), which can be seen in Figure 11.

## 5. Conclusions

Biocomposites in granules were obtained from industrial waste tyre rubber, fly ash cenospheres, and homogenised peat.

As the amount of CR in the composite increases, its surface hydrophobicity increases, as evidenced by changes in the water wetting angle Θ from 95 to 152°. When applied to the composite CS, the wetting angle tends to decrease, but in no case is it less than 120°, which is typical for hydrophobic surfaces.

As the concentration of CR in the composite increases, the mechanical strength of the granules decreases from 48 N on average if the composition contains 5% CR to 26 N if the composition contains 30% CR. The incorporation of CS into the composition further reduces the mechanical compressive strength of the granules, which may be due to insufficient bonding of the microspheres and the homogenised peat, on the one hand, and a high degree of composite-filling defects and voids, on the other.

The obtained granulated biocomposites based on HP, CR, and CS are characterised by a sufficient capacity to bind petroleum products: diesel fuel and motor oil. With the increase of the CR content in the granules, the absorption capacity of diesel fuel increases significantly on average from 0.5 g·g^−1^ in the case of homogenised peat to 1.3 g·g^−1^ for granules with the composition 30-70-0. The increase in the binding capacity can be explained by two factors: the clearly expressed hydrophobicity of the surface and the developed porous structure of the granules. External diffusion processes determine diesel binding to the granule surface and the diffusion processes of diesel fuel into the granule’s porous structure.

As the amount of CS in the granules increases up to 11.3 wt.% (for a dry composition), the DF absorption capacity increases significantly up to 0.7 g·g^−1^ sorption capacity for MO and up to 1.55 g·g^−1^ sorption capacity for DF (30-65-5), which is almost three times higher than the granules from the homogenised peat. The increase in q is due to two factors: the pronounced surface hydrophobicity of the samples (Θ = 152°) and a heterogeneous porous granule structure. The presence of CS in the composition of biocomposites reduces their hydrophobicity. However, due to high porosity, the binding capacity of diesel fuel is not almost reduced, while the buoyancy of the granules is increased.

In the case of motor oil, it was observed to bind mainly only by the surface of the granule and the maximum rate of binding was reached in the first minutes of the experiment. Increasing the CR and CS content in the biocomposite increases the binding capacity of motor oil due to the expanded structure on the surface of the granule.

The obtained pellets are characterised by a sufficiently pronounced ability to absorb MO and DF. Relatively quick (less than 10 min) achievement of the maximal saturation by the MO was noted. The presence of the cenosphere in the biocomposite reduces its surface hydrophobicity while increasing the DF absorption capacity.

## Figures and Tables

**Table 1:** Examples of developed and studied sorbents for crude oil and oil products’ spill clean-up and water treatments.

Adsorbent Production Method, Composition	Adsorbate	Adsorption Capacity g·g^−1^	Form, Shape	Ref.
Plant fiber foam hexamethyldiazosilane + NH_3_ vapours treatment	Olive oil	38	Sponge	[7]
WTR graft copolymerisation-blending method, 4-tert-butylstyrene + divinylbenzene, and benzoyl peroxide	Crude oil (10%) diluted with toluene	24	Spherical beads 0.5–1 mm in diameter	[8]
Graphene aerogel	Emulsified diesel oil	25	Aerogel sponge	[12]
WTR activated through carbonisation and H_2_SO_4_ treatment	Crude oil	14.7	Powder	[13]
Reduced graphene oxide and natural rubber latex	Crude oil	12–21	Aerogel sponge	[15]
Peat activated through carbonisation at K_2_CO_3_ presence	Crude oil	7–8	Powder	[14]
Natural peat, no treatment	Motor oil	2.1–6.5	Bulk peat fibers, 1–7 mm	[16]
WTR powder	Crude oil	4.7	Powder	[17]
Rice straw, no treatment	Water/Diesel mixture	1.8–2.3	Bulk straw	[18]
Expanded perlite	Petroleum products	2–2.5	Powder	[19]
Cellulosicfibre		2–3.8	Bulk fibers	
Straw sorbent, no treatment	Water/Oil product mixture	0.033	Bulk straw	[20]
Peat sorbent, no treatment		0.037	Bulk peat	
Mineral sorbent (cemetious residue), no treatment		0.00183	Powder	

**Table 2:** The composition of block and granules in the raw mixture (wet) and after drying by wt.% (part I) [4].

	Designation of the Composition											
	0-100-0	5-95-0	10-90-0	15-85-0	20-80-0	30-70-0	0-95-5	5-90-5	10-85-5	15-80-5	20-75-5	30-65-5
**Wet mixture composition (wt.%)**												
DCR	0.0	5.0	10.0	15.0	20.0	30.0	0.0	5.0	10.0	15.0	20.0	30.0
HP	100	95.0	90.0	85.0	80.0	70.0	95.0	90.0	85.0	80.0	75.0	65.0
CS	0.0	0.0	0.0	0.0	0.0	0.0	5.0	5.0	5.0	5.0	5.0	5.0
	**Dried composite material formulation (wt.%)**											
DCR	0.0	27.3	44.2	55.8	64.1	75.4	0.0	22.1	37.2	48.1	56.3	68.0
HP	100	72.7	55.8	44.2	35.9	24.6	72.7	55.8	44.2	35.9	29.6	20.6
CS	0.0	0.0	0.0	0.0	0.0	0.0	27.3	22.1	18.6	16.0	14.1	11.3

**Table 3:** The composition of block and granules in the raw mixture (wet) and after drying by wt.% (part II) [4].

	Designation of the Composition					
	0-90-10	5-85-10	10-80-10	15-75-10	20-70-10	30-60-10
**Wet mixture composition (wt.%)**						
DCR	0.0	5.0	10.0	15.0	20.0	30.0
HP	90.0	85.0	80.0	75.0	70.0	60.0
CS	10.0	10.0	10.0	10.0	10.0	10.0
	**Dried composite material formulation (wt.%)**					
DCR	0.0	18.6	32.1	42.3	50.3	62.0
HP	55.8	44.2	35.9	29.6	24.6	17.4
CS	44.2	37.2	32.1	28.2	25.1	20.7

**Table 4:** Influence of CS on the properties of biocomposites in the shape of a block.

Parameter	CR 0 wt.%			CR 5 wt.%			CR 10 wt.%		
	CS 0 wt.%	CS 5 wt.%	CS 10 wt.%	CS 0 wt.%	CS 5 wt.%	CS 10 wt.%	CS 0 wt.%	CS 5 wt.%	CS 10 wt.%
σ, MPa	79.0	2.2	1.7	7.7	2.0	1.8	5.5	1.3	1.1
ρ, g·cm^−3^	1.35	0.74	0.64	1.20	0.76	0.65	1.10	0.85	0.70
Θ, ^°^	95	115	124	135	121	129	139	122	132

**Table 5:** Apparent density, porosity, and specific surface area of biocomposite granules of different compositions.

Granule Composition	Apparent Density ƍ, g·cm^−3^	Porosity, %, BET	Porosity, %, (by MCt)	Specific Surface Area, m^2^·g^−1^
0-100-0	1.29	17.8	19.7	0.452
5-95-0	1.20	32.9	34.7	0.580
5-90-5	0.71	37.6	39.5	0.702
